# Formononetin could increase soluble-APPα secretion by up-regulating ADAM10 level

**DOI:** 10.1186/1750-1326-7-S1-O10

**Published:** 2012-02-07

**Authors:** Ting Zhou, Miao Shun, Liang Zhou, Huan Yang, Kaiyin Zhong, Ximeng Zhang, Hui Zhang, Dongsheng Fan, Dehua Chui

**Affiliations:** 1 Peking University Neuroscience Research Institute, Dep. of Neurology, Peking University Third Hospital, Beijing 100191, China

## Background

Formononetin, which is used as neuroprotective medicine, was reported to have benefits for Alzheimer's disease (AD). However, little is known on how Formononetin exerts these beneficial effects. In this study, we investigated the molecular mechanisms through which Formononetin increased soluble-APPα (sAPPα) secretion and thus was neuroprotective in human-APP Swedish mutation cell cultures (N2a-APP cell).

## Method and results

By using N2a-APP cell cultures combined with hypoxia treatment, we confirmed that chronic treatment with Formononetin could have neuroprotective effects, which was followed by reduced and increased Caspase3 activity and cell viability. Strikingly, our data revealed that the Caspase3-blocking effect of Formononetin was largely mediated by stimulation of α-secretase cleavage of APP, resulting in increased secretion of its soluble form, sAPPα. Moreover, the protective effect of Formononetin was totally inhibited by TAPI-2, an α-secretase complex inhibitor, suggesting the role of the sAPPα pathway in the neuroprotective response to Formononetin. Furthermore, we also revealed that the stimulation effect of Formononetin on α-secretase activity was mainly a result of up-regulating ADAM10 expression at the transcriptional level.

## Conclusion

Altogether, our study provides novel insights into how Formononetin mediated stimulation of the ADAM10-sAPPα pathway and resulting neuronal protective effect.

